# How effective and sustainable is proctoring in robotic surgery? A retrospective analysis based on interviews with surgeons

**DOI:** 10.1007/s00464-024-11503-5

**Published:** 2025-01-30

**Authors:** Veronika Günther, Frauke Nees, Nicolai Maass, Sören von Otte, Zino Ruchay, Julian Pape, Johannes Ackermann, Ibrahim Alkatout

**Affiliations:** 1 Department of Obstetrics and Gynecology, University Hospitals Schleswig-Holstein, Campus Kiel, Arnold-Heller-Strasse 3 (House C), 24105 Kiel, Germany; 2 University Fertility Center, Ambulanzzentrum des UKSH gGmbH, Arnold-Heller-Strasse 3 (House C), 24105 Kiel, Germany; 3 Institute of Medical Psychology and Medical Sociology, University Medical Center Schleswig-Holstein, Kiel University, Preusserstrasse 1-9, 24105 Kiel, Germany

**Keywords:** Proctoring, Robotic surgery, Feedback, Learning needs, Surgeons, Sustainability

## Abstract

**Background:**

Robot-assisted surgery is one of several minimally invasive techniques which have become increasingly important in recent years. Education and training are key factors of sustainable success, and surgical tutoring by an experienced external surgeon (proctoring) has emerged as a very useful method of training surgeons. Proctoring enables surgeons to train their respective skills and eventually improve the overall quality of surgical care.

**Methods:**

To evaluate the sustainability of proctoring, we conducted telephone interviews with colleagues who participated in a single-center surgical proctoring program. The aim was to analyze the feasibility of the concept for being established in the future, how well it suits the surgeons (depending on their individual work experience), and which areas of clinical practice would benefit from proctoring. Furthermore, the surgeons provided free-text comments to the questions on a feedback form handed out after the proctoring.

**Results:**

Surgical skills were significantly enhanced and operating times were reduced after proctoring. Given favorable structural conditions, the number of cases and the case-mix index were also significantly increased. It was found that the learning needs of surgeons differ, depending on their medical function, work experience, and the frequency of robotic surgery. On the feedback forms, proctoring was consistently rated as a positive and supportive measure.

**Conclusions:**

Proctoring is an important aspect of the individual development of surgeons. Depending on learning needs, a structured curriculum is crucial and should be established for the future. Regular feedback modules should be a part of any surgical training concept.

**Supplementary Information:**

The online version contains supplementary material available at 10.1007/s00464-024-11503-5.

Minimally invasive techniques have become increasingly important in recent years, particularly in the fields of visceral and thoracic surgery, urology and gynecology [1]. The range of surgical techniques, including robot-assisted surgical methods, which have been consistently developed over the past few years, offer patients the option of differentiated and individualized surgical treatment while fulfilling the standards required of modern medicine [2, 3].

These advancements present significant challenges: firstly, the integration of new and innovative techniques into clinical practice; secondly, ensuring that junior surgeons are trained in a structured, expert-guided manner. This allows them to safely apply these new surgical techniques, manage potential complications with confidence, and adhere to the ethical principle of non-maleficence, ensuring they cause no harm while advancing their skills [4, 5].

Most of the robotic systems have a simulator and have training instruments that allow the surgeon to practice before operating room and train and retrain himself before robotic operation. Evaluation of this simulator training has shown that with structured training and proctoring the results of the trainee could be similar with the trainer in structured supervised training [6]. Comparing this simulator training to other fields such as aviation, where realistic training can be conducted on a simulator, we lack a developed method of really true-to-life training in robot-assisted surgery. As the procedures necessarily include the stress factor, the training must take place in actual patient situations. A significant portion of the learning curve is traversed during the treatment of patients, and this raises ethical concerns [7]. Animal or human cadaver models are inadequate compromises because their applicability is limited to specific aspects of training. The large majority of clinics in Germany lack the financial resources, and/or time needed for prolonged clinical leave. Cross-center training through so-called proctoring has emerged as a form of in-house training for surgeons in robot-assisted surgery. The concept of “flying doctors” may be viewed as a preliminary stage [4, 8]. Surgical tutoring by an experienced external surgeon enables hospitals to extend and refine the skills of their in-house surgeons, and thus improve the overall quality of treatment [9]. Proctoring is valuable even for experienced doctors, as some may be unfamiliar with new techniques. It helps them adapt to innovations by providing structured guidance. The measurable outcomes of proctoring include improvements in operating times and patient transfers, as well as a reduction in complication rates, ensuring safer and more efficient surgical practices [10].

A refinement of the proctoring program would reinforce the critical learning phase: the proctor uses a special feedback sheet and provides the trainee with additional and sustainable feedback after the proctoring has been concluded. The surgeon is then able to implement the tailored suggestions and share these with his/her surgical team [5]. The concept of a single proctoring session was expanded into a series of feedback sessions, offering the trainee continued support and guidance. However, we lack a program of regular training and feedback in clinical routine for surgeons with limited, as well as those with several years of professional experience. Proctoring could be a part of the surgical curriculum for the entire duration of a surgeon’s professional life. This study aims to retrospectively collect data through telephone interviews to assess the benefits and sustainability of proctoring in robot-assisted surgery. Specific attention is given to individual learning needs and work experience. Furthermore, we evaluated the personal advantages of the newly established feedback form with answers provided by the respondents as free-text comments.

## Material and methods

Since November 2022, an experienced and accredited robotic surgeon has been proctoring in the field of robot-assisted laparoscopy in gynecology. Medical professionals received support in their own clinic, benefiting from the proctor’s long-standing surgical experience. The proctoring comprised one day, during which the proctor continuously accompanied the operational team to be trained. The number of joint operations varied depending on the duration and degree of difficulty of the operation, and ranged from one to four operations. Oncological, surgically demanding procedures were often chosen by the trainees in order to further increase the benefits of proctoring—also for the patients. Particularly in clinics where robotic surgery was newly introduced, the learning requirement was often higher, so that sometimes up to four proctoring appointments took place within a year.

Intraoperatively, both the surgeon (mostly senior physicians or chief physicians) and the table assistant, as well as the anesthesia and the surgery nursing staff, received direct feedback from the proctor with suggestions for corrections and improvements. In the event of a difficult surgical situation, it was also possible for the proctor to take over the operation himself for a short time.

To date, proctoring has been performed in about 30 centers in Germany, for exampleHamburg, Berlin, Greifswald, Cologne, Switzerland (Bern), and France (Dijon), etc. After the proctoring session, the surgical team received a detailed feedback sheet via e-mail, which summarized the day`s events in detail and addressed the strengths and weaknesses of the respective surgeons. The feedback form is included in the Supplemental Data of this report.

We conducted telephone interviews with medical professionals who attended the proctoring program in order to evaluate the sustainability of proctoring and the doctors’ learning needs in robotic surgery. We gave preference to telephone interviews rather than questionnaires because of the strong methodological value of the former. Higher response rates, ease of implementation, greater complexity, a preference for extreme response categories, and greater depth of response are some of the advantages of telephone interviews over paper–pencil questionnaires or online surveys [11, 12]. On the other hand, there might be a potential bias due to self-reported data through telephone interviews. It could be that the interviewees do not answer exclusively neutrally, but formulate the questions much more positively due to the personal level during the interview.

We tried to avoid this bias by taking the following measure: all interviews have been done by one clinical consultant (V.G.) who was not involved in the proctoring process nor personally known to the interview partners. Furthermore, the proctoring program was standardized across the centers. All the proctorings involved the same proctor, i.e., the way in which the operations were trained, the focus, and know-how, as well as the subsequent feedback were the same in each case (leaving aside the different operations and knowledge of the operators). The aim of this study was to analyze how promising the current concept is (including the feedback form), how well it suits the surgeons in relation to their individual work experience and medical position in the clinic, and whether it promotes the personal development of the trained doctor. The interviews were conducted from September 2023 to March 2024. A total of 40 surgeons who attended the proctoring program were interviewed.

The questionnaire comprised two sections, each consisting of six questions, designed to evaluate the personal benefits and sustainability of proctoring as well as individual learning needs. The questions were answered on a scale from 1 to 6, with 1 indicating that the statement was fully applicable and 6 indicating that it was not applicable at all. The questionnaire used for the telephone interviews, along with the rating scale, is included in the Supplemental Material. Finally, the surgeons provided free-text responses about their personal benefits from proctoring and identified areas in clinical routine that would benefit from proctoring. Additional data were collected, including gender, age, academic qualification (none, doctor, assistant professor, professor), medical function in the clinic (specialist, senior physician, chief physician), professional experience, frequency of work as a robotic surgeon, number of proctoring sessions attended, the duration of proctoring, and the institutional affiliation (proprietorship) of the respective clinic (public, church, private). The questionnaire is included in the Supplemental Data. The ethics committee of the Medical Faculty of the Christian-Albrechts University of Kiel (Arnold-Heller-Str., House no. 9, 24,105 Kiel, Germany) approved the study (Vote no. D 548/23). Informed consent was obtained from all participants before their inclusion in the study.

### Statistical analysis

Gender, age, function, academic qualification, institutional proprietorship, and frequency of work as a robotic surgeon were examined as independent group variables with regard to their effect on the selected target variable using analyses of variance (ANOVAs). Quantitative values are presented as means and standard deviations. Pearson’s correlations were used to determine the association between the questionnaire items and work experience as well as the duration of proctoring. The level of significance was set to *p *≤ 0.05 and 2-sided tests were performed. The statistical software R, version 3.6.3, was used for calculation.

## Results

Forty surgeons (21 men and 19 women) were interviewed on the phone and included in the retrospective study. Their mean age was 46.8 (SD 7.9) years. Table 1 summarizes the above mentioned characteristics of the surgeons.

**Table 1:** Surgeons’ characteristics

Age (years)	
Mean (sd)	46.8 (7.9)
Gender	
Men	21
Women	19
Professional experience (years)	
Mean (sd)	20.1 (7.8)
Title (total/ percent)	
No title	5 (12.5)
Doctor	22 (55)
Assistant professor	3 (7.5)
Professor	10 (25)
Function in the clinic (total/ percent)	
Specialist	1 (2.5)
Senior physician	22 (55)
Chief physician	17 (42.5)
Frequency of use as a robotic surgeon (total/ percent)	
Once a week	31 (77.5)
Every two weeks	5 (12.5)
Twice a week	4 (10)
Number of proctoring appointments	
Mean (sd)	1.3 (0.7)
Duration of proctoring (hours)	
Mean (sd)	5.6 (3.4)
Bearer of the clinic (total/ percent)	
Public	29 (72.5)
Church	7 (17.5)
Private	4 (10)

*sd* standard deviation

The majority of the surgeons were able to improve the quality of their work through proctoring (40% and 45%; 1 and 2 on the response scale). After the proctoring period they were able to apply what they had learned (45% and 50%; 1 and 2 on the response scale).

All of the participating surgeons considered proctoring to be appropriate in relation to their previous experience in robotic surgery (80% and 20%; 1 and 2 on the response scale).

**Fig. 1:**
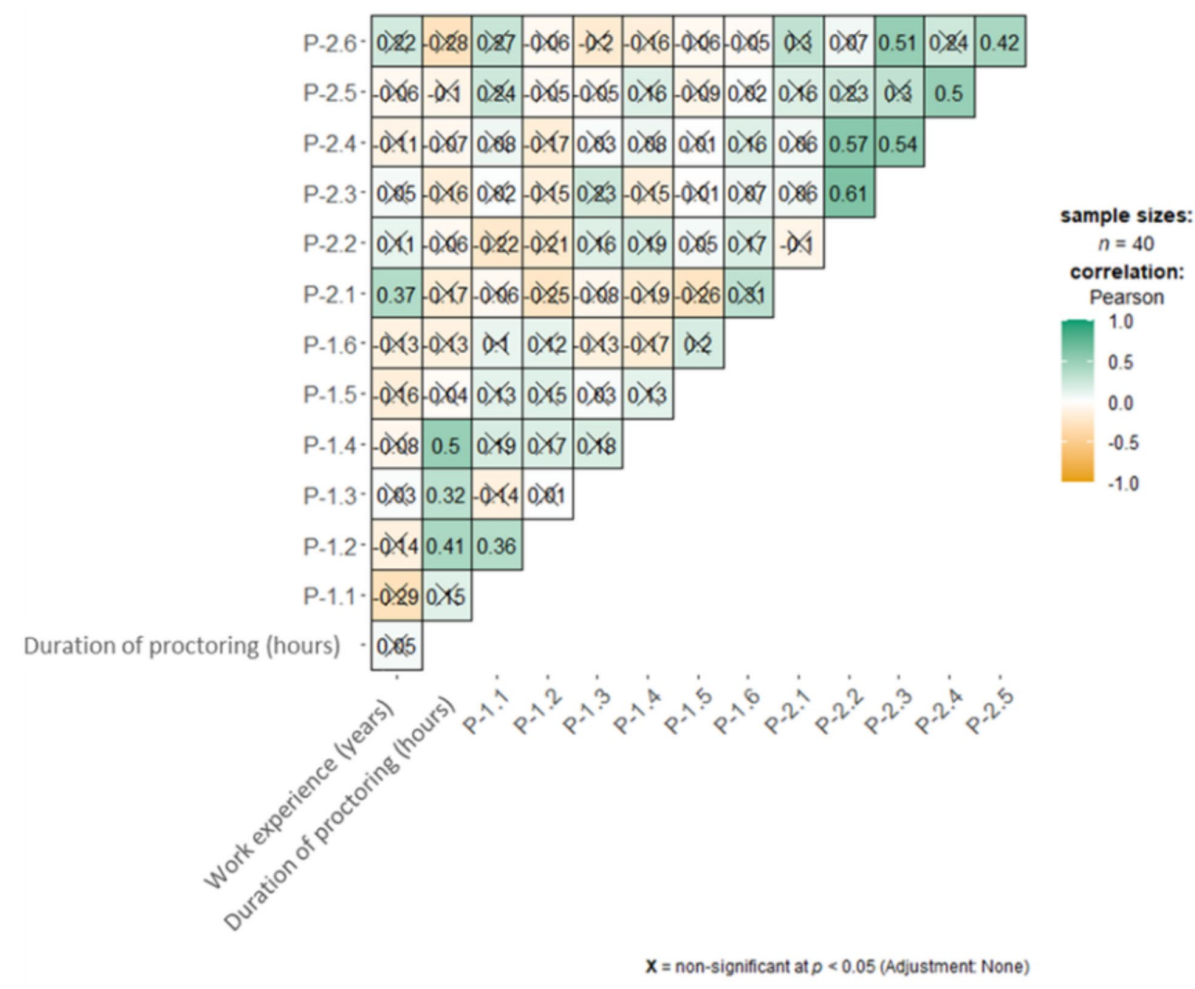
Heat map showing the associations between questionnaire items and work experience as well as the duration of proctoring, calculated with Pearson’s correlation. A significance level of p ≤ 0.05 was defined and 2-sided tests were performed. Statistically significant results are marked with a positive sign and are shown in green. The mirrored side of the square was omitted for a better overview. The crossed-out boxes are not statistically significant (p > 0.05). Significant correlations were found between work experience and assessment of the value of proctoring in conventional laparoscopy. In addition, the duration of proctoring was correlated with the need for learning, feedback, and its communication

Figure 1 is a heat map providing a general overview of correlations between work experiences, the duration of proctoring, and the answered questions. The individual associations are discussed in greater detail below.

**Fig. 2:**
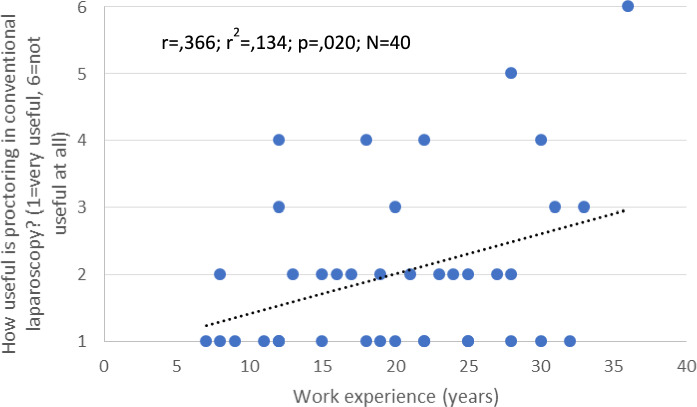
Proctoring in conventional laparoscopy. The more professional experience a surgeon had, the less useful he/she rated proctoring in conventional laparoscopy (p = 0.02)

Surgeons were asked to evaluate the usefulness of proctoring in conventional laparoscopy. The results indicated that as surgeons gained more professional experience, they found proctoring in conventional laparoscopy to be less beneficial (*p* = 0.02) (Fig. 2).

### Learning needs

**Fig. 3:**
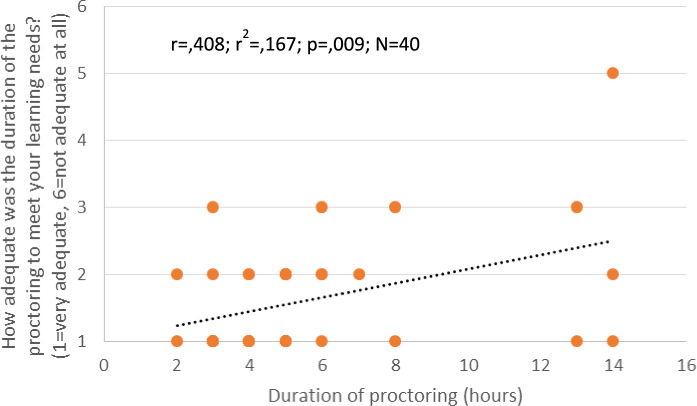
Duration of proctoring and learning needs. The longer the duration of proctoring, the less appropriate it was rated in terms of learning needs, i.e., the surgeons felt their learning needs had not yet been met, p = 0.009

The learning needs of the surgeons were determined regarding several variables. Question 1.2 inquired as to how appropriate the duration of the proctoring was in terms of meeting one’s own learning needs. The following response behavior emerged: the longer the duration of proctoring, the less appropriate it was deemed in terms of learning needs. In other words, the surgeons felt that their learning needs had not yet been met and would have liked to spend more time training (*p* = 0.009, Fig. 3).

The answers differed, depending on the frequency of work as a robotic surgeon. Three groups were formed: those performing robotic surgery once a week, every two weeks and twice a week. Surgeons who performed robotic surgery more often (specifically twice a week) rated the duration of proctoring as being more appropriate in terms of fulfilling their learning needs than those who performed robotic surgery less often (once a week) (*p* = 0.0019).

**Fig. 4:**
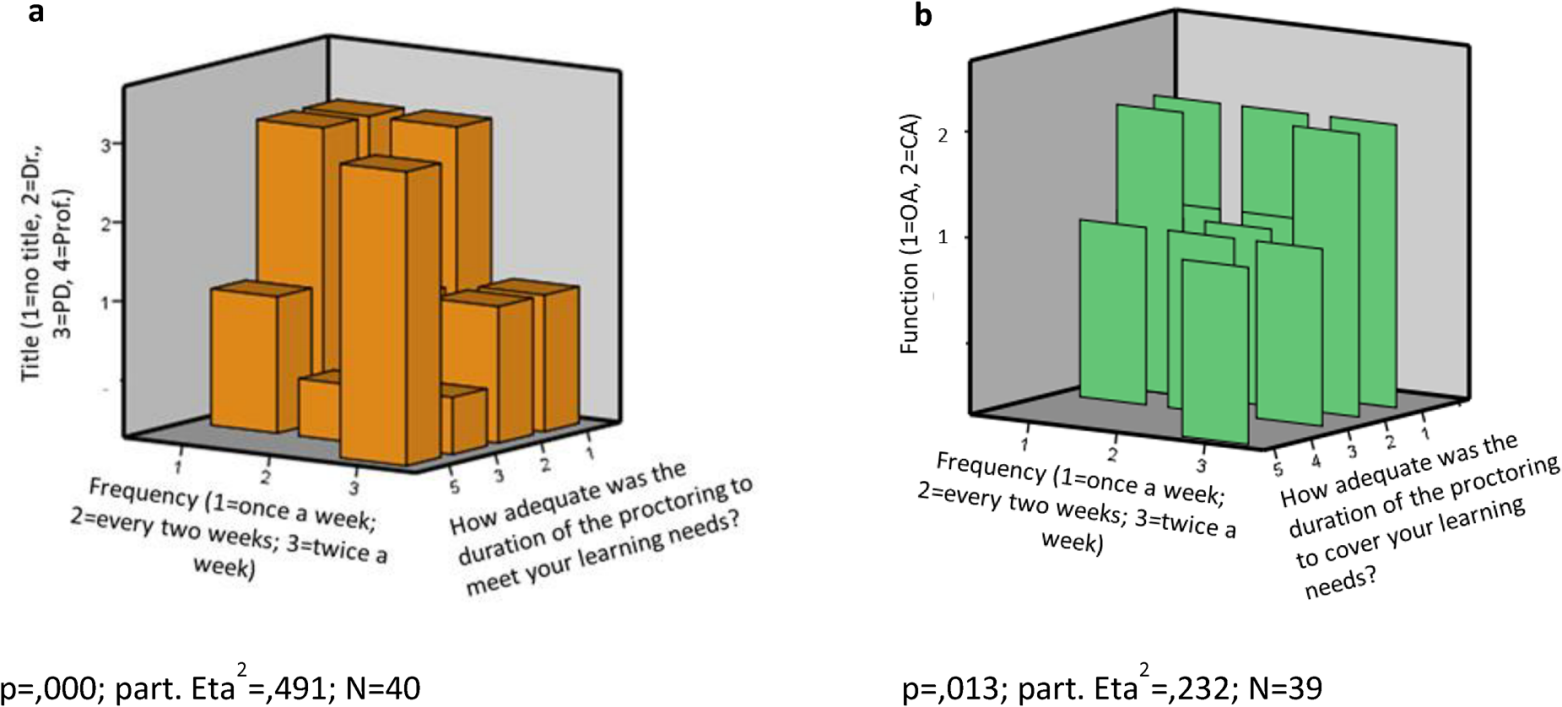
**a**: Learning needs depending on the frequency of robotic surgery and academic qualifications. Surgeons with a higher academic qualification who did not perform robot-assisted procedures frequently rated the duration of proctoring as being more adequate. **b**: Learning needs depending on the frequency of robotic surgery and medical position. Surgeons in a higher position (chief physician/ CA > senior physician/OA) who performed robot-assisted procedures more frequently rated the duration of proctoring as being adequate in terms of meeting their learning needs

The duration of proctoring in regard of fulfilling learning needs was rated differently also in relation to the academic qualification of the respondent: surgeons with a higher academic qualification (professor, assistant professor, doctor, in descending order) and those who did not perform robot-assisted procedures frequently gave the duration of proctoring a higher rating (*p* = 0.000, Fig. 4a). Surgeons in a higher position (chief physician, senior physician) and those who performed robot-assisted procedures more frequently rated the duration of proctoring as being more adequate in terms of fulfilling their learning needs (*p* = 0.013, Fig. 4b).

### Operating times and patients benefiting from proctoring

The impact of proctoring on operating times varied based on the surgeon’s frequency of performing robotic surgery and their academic qualifications. Surgeons with higher academic qualifications who performed robot-assisted procedures less frequently reported that proctoring significantly helped in reducing operating times (*p* = 0.047). Overall, 67.5% of the surgeons indicated that they were able to reduce their operating times following proctoring, giving a rating of 1 or 2 on the response scale. In all 92.5% of the surgeons stated that the patients benefited from proctoring (1 and 2 on the response scale). The extent of patients benefiting from proctoring was assessed differently, depending on academic qualifications and institutional proprietorship: Surgeons with higher academic qualifications who worked in public institutions stated that patients benefited less from proctoring (*p* = 0.013). Furthermore, the extent to which patients benefited from proctoring was also rated differently in relation to the surgeon’s medical function and institutional proprietorship: surgeons who held a higher position (chief physician, senior physician) and worked for church organizations stated that patients are more likely to benefit from proctoring (*p* = 0.038).

### Feedback

The surgeons received a feedback form that summarized the proctoring in detail. The majority of the surgeons considered feedback very important (92.5% and 7.5%; 1 and 2 on the response scale). Communication of the feedback during proctoring was rated excellent (87.5% and 12.5%; 1 and 2 on the response scale).

The feedback form helped most surgeons very markedly in applying the contents of proctoring to their clinical work (82.5% and 17.5%, 1 and 2 on the response scale). Differences were noted in regard of institutional proprietorship and the surgeon’s medical function: persons in higher positions working for church organizations considered the feedback form less helpful (*p *= 0.028).

### Influence of proctoring on the number of cases and the case-mix

**Fig. 5:**
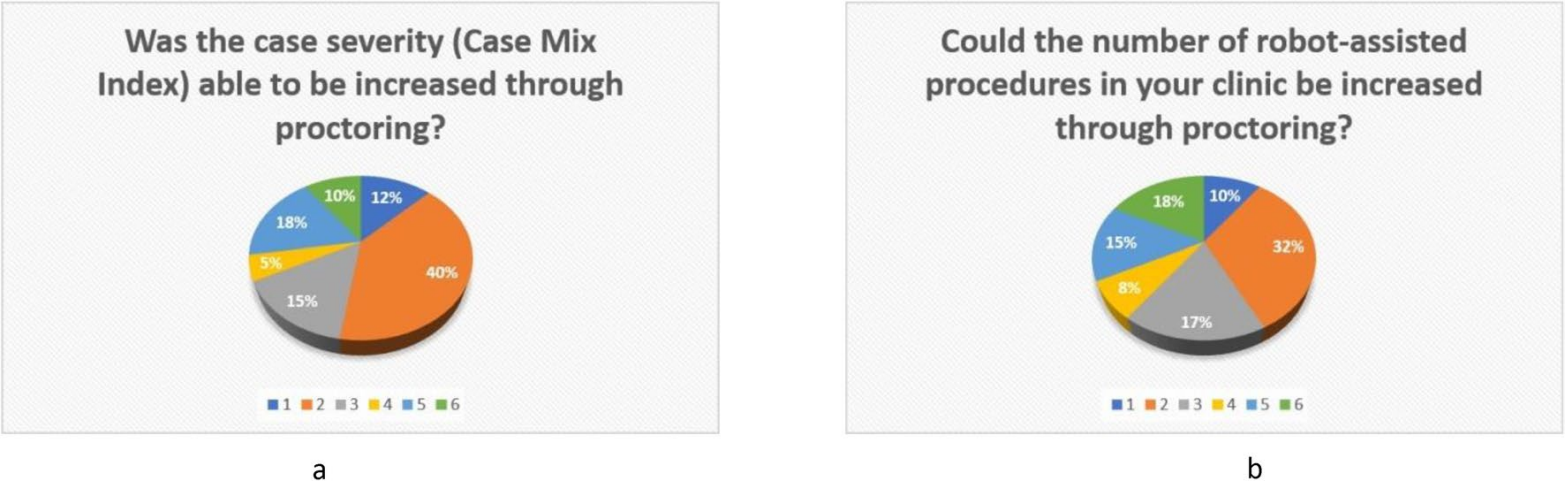
**a**, **b**: Response behavior (response scale 1–6) to the question as to whether the number of cases (**a**) and the case-mix index (**b**) could be increased through proctoring

The surgeons were asked whether proctoring had increased the number of robot-assisted procedures they performed and the severity of cases or the case-mix index. Figure 5a and b show the respective responses. All surgeons who rated this question 3 or worse on the response scale were asked whether their rating was related to the structure of the clinic, i.e., the number of cases could not be increased further due to the given framework conditions. This question was *answered in the affirmative* by 100% of the subgroup.

Finally, the surgeons described, in free-text, their personal benefits from proctoring. Table 2 shows some of their answers.

**Table 2:** Free-text comments from surgical colleagues who took part in the proctoring

What personal benefits have I gained from proctoring	In which areas of everyday clinical practice would it also make sense to introduce proctoring?
Great benefit from proctoring, systematic overview, good tips and tricks, e.g. for docking, overview of the spatial conditions, "mediator", neutral suggestions from an expert, psychological moment	Every area of gynecology and obstetrics, e.g. breast surgery, forceps/ BEL/ Gemini, similar to perineal protection learned by midwives
Good experience, use of expertise, sustainable changes in operational handling	Everywhere, necessity of process optimization, but first question of cost efficiency
Safety with a new technology, reducing reservations, optimization of processes in the operation room	In all areas where new methods are implemented, where complicated technology is introduced, with new devices. Cave: cost-intensive
Presentation and learning of a basic system. Gaining security	Urogynecology, for oncologically challenging cases, in prenatal medicine
Collegial connection, personal conversation, important exchange, focus on details	All areas, including management and at a structural level
Setting impulses, contact with other clinics, self-reflection, "thinking outside the box", exchange, experience	All operational areas, or where manual dexterity is required
Good opportunity to reflect on operational skills, risk awareness, strengthening of self-esteem	Anywhere where mistakes can happen
Experience and safety on site, "driving school" principle, 1:1 support	Everywhere in the implementation of new procedures, both diagnostic and therapeutic

## Discussion

The present investigation revealed that trained doctors benefited enormously from proctoring in terms of improving the quality of their work and operating times, and were able to transfer the surgical skills acquired during proctoring to clinical routine. In addition, it was found that learning needs are subject to individual differences and depend especially on work experience, medical position, academic qualifications, and the frequency of robotic surgery. Feedback plays an extremely important role and has been underestimated or given too little attention in the past.

Our perusal of the published literature on proctoring and surgery revealed very few studies on evaluations and outcomes of proctoring. Some authors investigated the effect of tele-proctoring, but we found scarce data on how proctoring is evaluated by doctors with different levels of professional experience and in different positions. Compared to other professions associated with performance, improvement and responsibility, such as aviation or competitive sports, we lack integrated, structured and routine feedback on interventions and operations in medicine. Thus, the methods of continuous improvement in medicine fail to meet the required standards. An appropriate supervision program would be highly desirable [13]. Ideally, it should include regular coaching during operations and subsequent feedback with a view to improving a surgeon’s skills.

We also lack data in the published literature about the evaluation of feedback after proctoring, especially the surgeon’s assessment of the importance and effect of proctoring. However, a large number of reviews address the teaching of robotic-assisted laparoscopic surgery in general, call for the implementation of guidelines, structured recommendations, the need for a curriculum with stepwise learning objectives, and regular access to a training robot [3, 14–18]. The demand for a curriculum is consistent with our data and the elicited individual learning needs. Especially when surgeons start to learn robotic surgery, they need a great deal of help and support to fulfil their learning requirements. The more often and the longer the proctoring was used (several appointments), the more support the surgeons needed in robotic surgery. Accordingly, the duration of proctoring was deemed insufficient to fulfil the surgeons’ learning needs.

On the other hand, those who frequently work as robotic surgeons rated the duration of proctoring as appropriate. Given their solid foundation in terms of surgical tools and practical skills, they needed no more than minor assistance. Robot-assisted surgery is still a relatively new surgical method [19]. All we can do at the present time is acquire sensitive data on its preliminary application. These data could give rise to ethical discussions or health policy issues. Once a procedure has been established, the fragile phase is concluded and the uncertainty of the new belongs to the past. One procedure that has successfully passed all of these phases is conventional laparoscopy.

Furthermore, we registered an improvement in surgical skills, greater self-confidence on the part of the surgeon (as mentioned frequently in the free-text comments), and shorter operating times. These data are consistent with those reported by Bowen et al. The latter group from Chicago analyzed the learning curve of surgeons in pediatric robotic-assisted laparoscopic pyeloplasty. The surgeons were very experienced in open surgery and converted to robotics accompanied by a proctor. Postoperative outcomes and operating times were evaluated to determine the learning curve. After proctoring, the radiological evaluation of pyeloplasty revealed a good outcome. In addition, a significant reduction in operating times was noted as a result of proctoring [10]. Similar data were reported by Murakami et al. [20]. Endoscopic surgery is not widely used in pediatrics, and this enhances the value of learning these procedures with the aid of a proctor. The authors reported that difficult procedures in endoscopy could be mastered with the aid of the proctor alone [20].

Feedback is a standard part of many professions, particularly in sensitive work fields. In aviation, pilots have to conduct training flights on the simulator even after decades of professional experience. However, feedback does not appear to play a visible role in medicine, particularly in surgery, although this profession is associated with a high level of responsibility and therefore calls for lifelong learning and improvement of skills [21]. Feedback plays an essential role in proctoring. At the beginning of their careers and even later on, many doctors are unable to separate critical feedback about a clinical action (performance) from their person. In addition, a doctor needs to develop strategies and strengthen his/her self-esteem in order to deal with critical feedback openly and appropriately. These aspects constitute an essential part of a doctor’s personal and professional development. Ideally, criticism should be viewed as an opportunity for growth.

Persons with a "growth mindset" have a strong intrinsic desire for improvement. They wish to develop their skills, improve their performance, and widen their professional spectrum [5, 22, 23]. Feedback in the context of proctoring has been underestimated or given too little attention in the published literature [5]. The present study showed that feedback was rated positively by all surgeons. Both, the intraoperative feedback and the post-proctoring feedback form supported the learning process and reinforced the acquired knowledge and skills.

A German study group divided medical students into a low- and a high-frequency feedback group while being trained on the use of a nasogastric tube [24]. The authors wished to determine how much feedback is needed to maximize the learning effect. All students were given structured feedback at the beginning of the course. The high-frequency feedback group received additional repetitive feedback after each of their five sections, whereas the low-frequency feedback group received additional feedback only after the fifth repetition. Task-specific clinical performance and global procedural performance were assessed at the beginning of the training and during the final sixth trial [24]. The authors concluded that both, high- and low-frequency intermittent feedback resulted in a strong improvement of the students’ early acquisition of procedural skills. High-frequency intermittent feedback, however, resulted in even better and smoother performance [24].

These data concur with those reported by Trehan et al. who showed, in a systematic review, that intraoperative feedback results in better performance, reduces procedure time, improves the economy of movement, and is associated with a smoother learning curve [25]. Precisely these points were also mentioned by the surgeons in the present study and described as positive factors in proctoring and feedback. Thus, it would be most desirable to extend perioperative feedback in terms of content, structure and quantity. In addition, surgeons should receive detailed feedback after completion of proctoring, as this would serve as a validation and appreciation of their skills.

Zorn et al. have addressed a specific problem concerning the safe initiation of robotic urological surgery [15]. The authors mention the absence of a standardized credentialing system to assess the doctor`s surgical skills at the commencement of his/her work in robotic surgery [15]. This is in strong contrast to other areas such as driving schools: the trainee is permitted to actively participate in road traffic only after he/she has passed the driving test. In aviation the pilot in training must undergo innumerable hours of training on the simulator before he/she is permitted to fly as a co-pilot [5]. In contrast to medicine or surgery, in aviation it is routine practice to correct and coach a pilot even after several years of professional experience [26]. These routine practices apply to pilots as well as their crew members. A similar setup would be most desirable in medicine: regular coaching, supervision, and being corrected for the purpose of improving one’s skills.

We advocate a structured curriculum for all surgeons who are new to the field of robotic surgery. Such curricula would be useful not only as a means of protecting doctors, but also serve the interests of patients [15]. The ethical angle is worthy of note. Although there is a simulator training program, there are sometimes considerable differences in the quality of the graphical representation, the feel of the instruments in the simulated tissue and thus the level of detail compared to real operations. Consequently, a part of the education has to take place directly in the operating room on humans. Even training programs on body donors or animal cadavers are no adequate substitute [27, 28]. This heightens the importance of proctoring, which offers at least some training and support for surgeons [29]. Pai et al. mention similar views in their report concerning medicolegal considerations on robotic surgery [30]. The authors address the absence of a standardized training program and credentialing for the surgeon`s competence on the one hand, and the patient`s safety on the other [30]. Especially the patient`s safety is an important factor when introducing new technologies. The quality of available studies concerning this topic was found to be low with current available evidence consisting largely of expert opinion, consensus statements and small qualitative studies. Improvements to training standards and understanding performance data have good potential to significantly lower complications in patients [31]. Pai et al. also draw attention to the patients’ awareness of robotic options and their potential risks; the authors emphasize the need for structured guidelines, regulations, and training programs to regulate the medicolegal aspects of robotic surgery effectively [30]. Shared decision-making (SDM) is a now a well-established healthcare delivery model in many clinics. SDM mandates patient-centered care in clinical practice. Practicing SDM ensures that patients and family caregivers are involved in making decisions about their care and treatment with healthcare providers [32]. The critical appraisal of shared decision-making (SDM) and transparency in proctoring highlights a potential dilemma. While transparency about surgeon training and case volume is essential for patient trust, it could deter patients from choosing newer procedures, especially when case numbers are low. This, in turn, may hinder the surgeon’s ability to gain experience, slowing the learning curve. The challenge is to balance transparency with promoting innovation; ensuring patients are well-informed while still supporting emerging surgical techniques and the proctoring process.

## Limitations

The study has a few limitations that will be mentioned in the following. First, there was no blinding in data collection, meaning the clinical consultant who did the telephone interviews was aware of the surgeons and their feedback; this may potentially introduce some bias, especially with telephone interviews. Second, key aspects such as the impact of proctoring on the learning curve, complication rates, hospital stay duration, and readmission rates were not evaluated, limiting the study’s scope.

Additionally, the sample size was relatively small, which may affect the generalizability of the results. The short duration of proctoring and different frequencies of proctoring sessions (from one up to four sessions), which was dependent on the request for a further proctoring appointment on the part of the senior physician of the clinic or the surgeon to be trained, also introduce variability in outcomes. The role of dual-console systems during proctoring sessions is another factor that has not been explored, yet may significantly influence surgical training.

In future studies, it would be beneficial to incorporate standardized proctoring protocols and assess the long-term effects of proctoring on clinical outcomes such as complication rates and patient recovery. Additionally, a larger sample size and extended follow-up would help validate the findings and provide a more comprehensive understanding of proctoring’s effectiveness in robotic surgery.

## Conclusion

The present investigation revealed that proctoring is an important part of a surgeon’s personal and professional development. The significance of proctoring on a one-to-one basis is heightened by the diverse learning needs of surgeons. The more difficult and complex an operation is and the less frequently it is performed, the more essential it is to have a proctor for learning the respective procedure. Surgical skills could be significantly enhanced and operating times reduced by proctoring. Given favorable structural conditions in the respective clinic, even case numbers and the case-mix index could be increased.

Feedback is closely linked to proctoring and is consistently rated as a positive and supportive learning element by surgeons. In proctoring, the feedback form serves as an individual curriculum and guideline for the future. It would be desirable to incorporate structured feedback processes into regular training modules throughout the professional career of a surgeon.

## Supplementary Information

Below is the link to the electronic supplementary material.

Supplementary file1 (PDF 187 KB)

Supplementary file2 (DOCX 21 KB)

## Data Availability

The datasets analyzed for the current review are available from the corresponding author on reasonable request.
