# Correction: How effective and sustainable is proctoring in robotic surgery? A retrospective analysis based on interviews with surgeons

**DOI:** 10.1007/s00464-025-11607-6

**Published:** 2025-02-10

**Authors:** Veronika Günther, Frauke Nees, Nicolai Maass, Sören von Otte, Zino Ruchay, Julian Pape, Johannes Ackermann, Ibrahim Alkatout

**Affiliations:** 1 Department of Obstetrics and Gynecology, University Hospitals Schleswig-Holstein, Campus Kiel, Arnold-Heller-Strasse 3 (House C), 24105 Kiel, Germany; 2 University Fertility Center, Ambulanzzentrum des UKSH gGmbH, Arnold-Heller-Strasse 3 (House C), 24105 Kiel, Germany; 3 Institute of Medical Psychology and Medical Sociology, University Medical Center Schleswig-Holstein, Kiel University, Preusserstrasse 1-9, 24105 Kiel, Germany

**Correction: Surgical Endoscopy** 10.1007/s00464-024-11503-5

The original online version of this article was revised to note that Open Access funding was enabled and organized by Projekt DEAL.

The original article has been corrected.

