# Supplementary material for: How effective and sustainable is proctoring in robotic surgery? A retrospective analysis based on interviews with surgeons
Source: Surg Endosc. 2025 Jan 30;39(3):1985–95. doi: 10.1007/s00464-024-11503-5 (PMC11870960; doi:10.1007/s00464-024-11503-5)
Supplement: Supplementary file 1 — Supplementary file1 (PDF 187 KB) [file 464_2024_11503_MOESM1_ESM.pdf]

Proctoring Feedback

ORT:

DATUM:

CHIRURG VOR ORT:

PROCTOR:

01 / Rahmenorganisation Intuitive

02 / Ärzte- und OP-Team vor Ort

a. Ziele

b. Selbsteinschätzung

c. Fremdeinschätzung / positiv

d. Fremdeinschätzung / kritisch

03 / Klinik

a. Zur Klinik

b. OP-Ablauf

Fall	Freigabe	Start OP	Pneumo-peritoneum	Docking	Start Konsole	Anzahl Instrumenten Wechsel	Setting Korrekturen	Ende Konsole	Ende OP
1.									
2.									
3.									

c. Tipps und Tricks

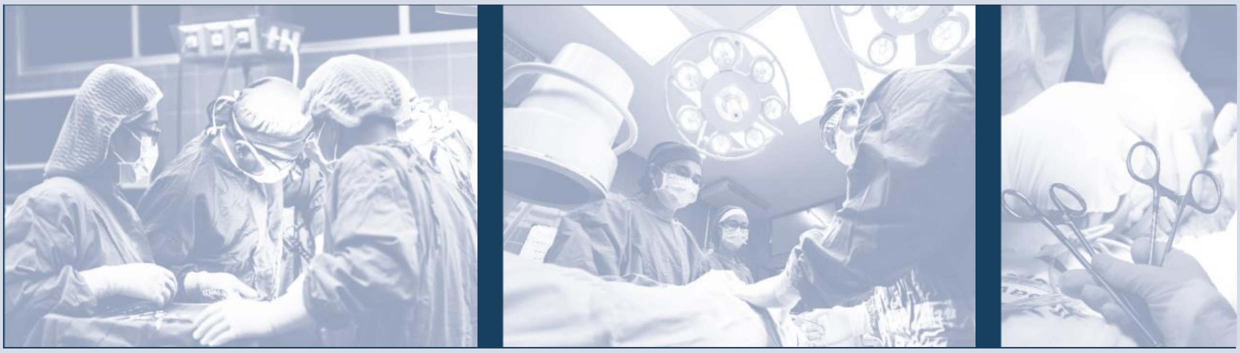

04 / Abschließende Zusammenfassung

Mit freundlichen Grüßen,

Prof. Dr. med. Ibrahim Alkatout M.A., MaHM
