# Supplementary material for: How effective and sustainable is proctoring in robotic surgery? A retrospective analysis based on interviews with surgeons
Source: Surg Endosc. 2025 Jan 30;39(3):1985–95. doi: 10.1007/s00464-024-11503-5 (PMC11870960; doi:10.1007/s00464-024-11503-5)
Supplement: Supplementary file 2 — Supplementary file2 (DOCX 21 KB) [file 464_2024_11503_MOESM2_ESM.docx]

**Questionnaire Follow-up Proctoring**

Name der Klinik:

Männlich/ Weiblich:

Alter:

Funktion in der Klinik:

Berufserfahrung (Jahre):

Ich bin als Roboterchirurgin/-chirurg x mal pro Woche im Einsatz:

Dauer des Proctorings (Zeitstunden):

Datum des Follow-up:

1. **Proctoring**
   1. Wie angemessen fanden Sie das Proctoring in Bezug auf Ihre Vorerfahrung in der Roboterchirurgie?
   2. Wie angemessen war die Dauer des Proctorings, um Ihren Lernbedarf zu decken?
   3. Wie wichtig ist Feedback für Sie in Hinblick auf das Proctoring?
   4. Wie gut wurde das Feedback während des Proctorings kommuniziert?
   5. Wie gut konnten Sie nach dem Proctoring die vermittelten Inhalte anwenden?
   6. Wie gut hat Ihnen dabei der Feedback-Bogen geholfen?
2. **Anwendung im Klinikalltag/ „Leistungskontrolle“**
   1. Wie sinnvoll ist ein Proctoring in der konventionellen Laparoskopie?
   2. Konnte die Fallzahl an Roboter-assistierten Eingriffen in Ihrer Klinik durch das Proctoring gesteigert werden?
   3. Konnte die Fallschwere (Case Mix Index) durch das Proctoring erhöht werden?
   4. Konnten durch das Proctoring die einzelnen OP-Zeiten verkürzt werden?
   5. Wie nachhaltig wurde durch das Proctoring Ihre operative Qualität verbessert?
   6. Wie gut profitieren die Patientinnen - auch unter Sicherheitsaspekten - von dem Proctoring?
3. **Weitere Bemerkungen und Kommentare (Freitext)**
   1. Welchen persönlichen Nutzen haben Sie aus dem Proctoring gezogen?
   2. In welchen Bereichen des klinischen Alltags ist es aus Ihrer Sicht sinnvoll, ebenfalls ein Proctoring einzuführen?

**Bewertungsskala**

Skala 1-6

__________________________________________________________________________________

1 (trifft voll zu) 6 (trifft überhaupt nicht zu)
